# Intranasal Administration of a Novel ApoE‐Mimetic Peptide‐Coated Gold Nanoparticles as Therapy for Ischemic Stroke

**DOI:** 10.1111/cns.70263

**Published:** 2025-03-18

**Authors:** Ming‐Yan Yang, Ya‐Wen Yu, Da‐Lei Li, Teng Liu, Zhi‐Xia Wang, Bai‐Fang Gong, Xin‐Xin Bai, Ya‐Ping He, Hai‐Yue Liang, Hua‐Ying Fan

**Affiliations:** ^1^ School of Pharmacy, Key Laboratory of Molecular Pharmacology and Drug Evaluation (Yantai University), Ministry of Education, Collaborative Innovation Center of Advanced Drug Delivery System and Biotech Drugs in Universities of Shandong Yantai University Yantai China; ^2^ Yantai Center for Food and Drug Control Yantai China

**Keywords:** ApoE mimetic peptide, gold nanoparticles, intranasal delivery, ischemic stroke, neuroinflammation

## Abstract

**Background:**

Discovering new drugs for ischemic stroke is an effective intervention that may address the significant unmet clinical need of stroke. There is increasing evidence indicating that apolipoprotein E (ApoE) can be a potential candidate for the treatment of ischemic stroke. A short ApoE peptide could maintain the anti‐inflammation and neuroprotection of the intact protein. Herein, we synthetized a novel ApoE memetic peptide, referred to as CS15, and explored its efficacy and neuroprotection of its innovative formulation of gold nanoparticles (GNPs) in transient focal ischemia in rat.

**Methods:**

We examined anti‐inflammatory activities of CS15 using LPS‐induced inflammatory response in BV2 cells and in mice. GNPs were prepared by citrate reduction method and surface modified with CS15 to generate CS15‐coated GNPs (CS15‐GNPs). The accumulation and distribution of CS15‐GNPs in the brain were confirmed by detecting the gold amount and fluorescent intensity. The neuroprotection of CS15 and CS15‐GNPs was evaluate using middle cerebral artery occlusion (MCAO) model.

**Results:**

The results showed that CS15 exhibited more potent anti‐inflammation than COG1410. GNPs are capable of transporting CS15 to the brain, expanding its duration of action. Intranasal administration of CS15‐GNPs notably reduced infarct size and neuronal damage, improved neurological function and inhibited cerebral inflammation in transient focal ischemia in rat, which had much higher efficiency than free CS15.

**Conclusion:**

CS15‐GNPs exhibited favorable neuroprotection and biosafety. This study develops an innovative ApoE‐mimetic peptide‐capped GNPs, which provides a potential strategy for the treatment of ischemic stroke.

## Graphical abstract

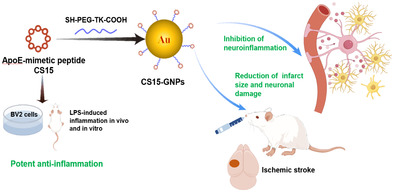

**Abbreviations**
ALT: alanine aminotransferase
ApoE: apolipoprotein E
AST: aspartate aminotransferase
BBB: blood–brain barrier
BUN: blood urea nitrogen
CCA: common carotid arteries
CFDA: China Food and Drug Administration
CK: creatine kinase
CNS: central nervous system
CRE: creatinine
CS15‐GNPs: CS15‐coated GNPs
DAPI: 4′,6‐diamidino‐2‐phenylindole
DBLK: piperidine/DMF solution
ECA: external carotid arteries
ED_50_
: median effective dose
EDC: 1‐ethyl‐3‐(3‐dimethylaminopropyl) carbodiimide
GNPs: gold nanoparticles
HPLC: high performance liquid chromatopraphy
HRP: horseradish peroxidase
IC_50_
: 50% inhibitory concentrations
ICA: internal carotid arteries
ICH: intracerebral hemorrhage
ICP‐MS: inductively coupled plasma mass spectrometry
LDLR: low‐density lipoprotein receptor (LDLR)
LPS: lipopolysaccharide
LRP1: LDLR‐related protein 1
MCAO: transient middle cerebral artery occlusion
NHS: N‐hydroxysuccinimide
TBI: traumatic brain injury
T‐BIL: total bilirubin
TEM: transmission electron microscopy
TFA: trifluoroacetic acid
TTC: 2,3,5‐triphenyltetrazolium chloride
UV–Vis: ultraviolet–visible

## 1 Introduction

Stroke remains the second leading cause of disability and death worldwide, in which ischemic stroke accounts for ~87% of all cases [1, 2]. During the past few decades, the global stroke burden increased substantially [1]. Despite advancing the treatment of ischemic stroke, the mainstay of treatment of ischemic stroke remains intravenous thrombolysis with alteplase [3]. To date, no neuroprotective agents have been approved by FDA for the treatment of patients with ischemic stroke.

Development of therapeutical agents from endogenous proteins and peptides as remedies for neurological disorders have attracted attention of the researchers. Apolipoprotein E (ApoE) is one of the key lipoproteins in regulation of the metabolism of lipids. The brain is the second major organ producing ApoE in addition to liver. ApoE is the most abundant apolipoprotein in the brain, which plays a significant role in the formation of synapses and myelination, neuronal proliferation, and regulation of neuroinflammation. ApoE can be a potential candidate for the treatment of central nervous system (CNS) diseases [4]. The naive ApoE is failed to penetrate the blood–brain barrier (BBB) due to its large size. Hence, designing short peptides with ApoE neuroprotective effects is a potential strategy for ischemic stroke. Currently, several ApoE memetic peptides have been developed and investigated in brain diseases clinically and experimentally, such as CN‐105, COG112, and COG1410 etc. There was evidence that these ApoE memetic peptides exerted anti‐neuroinflammation, antiexcitotoxic and neuroprotective effects in translational models of CNS disorders [5, 6, 7, 8, 9, 10].

Clinical application of peptide drugs is hampered by their intrinsic physicochemical and biological properties, including poor permeability and stability through gastrointestinal tract, metabolic degradation and short half‐life, resulting in poor bioavailability, and inferior efficacy. These render delivery of peptide drugs highly challenging [11]. Gold nanoparticles (GNPs) are a class of ideal nanomaterial. In recent years, they are extensively studied because of their potential biomedical applications in bioimaging, drug delivery, and therapeutics. GNPs possess unique properties, including excellent biocompatibility, small size and large surface area, versatile surface functionalization, and ease to synthesis [12]. Small sized‐GNPs easily cross the BBB, enabling targeted drug delivery to the brain. The large surface area provides ample space for more therapeutic agents to be attached to the surface. Surface modification allows GNPs to be easily functionalized with specific biomaterials such as ligand or peptides [12]. These make GNPs a promising solid platform for delivering of oligonucleotides, peptides, and proteins.

Given that GNPs can enhance the efficacy of biotherapeutics, in the current study, we synthetized a novel ApoE memetic peptide, referred to as CS15, and conjugated it to GNPs. We describe herein the synthesis of CS15 and its anti‐inflammatory activities in vitro. Furthermore, we will report the preparation of CS15‐covered GNPs and explore the neuroprotection of the gold formulation in transient focal ischemia in rat.

## 2 Material and Methods

### 2.1 Chemical and Regents

SH‐PEG2000‐TK‐COOH was purchased from Shanghai Pengsheng Biotechnology Co. Ltd. (Shanghai, China). CY5‐PEG2000‐SH was purchased from Xi'an Qiyue Biotechnology Co. Ltd. (Xi'an, China). NeuN antibody was obtained from Boster (BM4354, Wuhan, China). One step TUNEL apoptosis assay kit, immunol fluorescence staining kit with FITC‐labeled goat anti‐rabbit IgG, horseradish peroxidase (HRP)‐labeled goat anti‐rabbit IgG were product of Beyotime Biotechnology (Shanghai, China). Rat/mouse TNF‐α and IL‐6 ELISA kits were products of Multi Sciences (Lianke) Biotechnology (Hangzhou, China). Lipopolysaccharide (LPS, *Escherichia coli* O55:B5, product no. L6529) and 2,3,5‐triphenyltetrazolium chloride (TTC, T8877) were products of Sigma. All other chemicals were of analytical grade.

### 2.2 Cell Line

Mouse microglial cells BV2 were purchased from cell bank of Chinese Academic of Sciences (Shanghai, China) and cultured in DMEM medium supplemented with 10% heat‐inactivated FBS, 100 U/mL penicillin, and 100 μg/mL streptomycin, in a humidified atmosphere of 5% CO_2_ and 95% air at 37°C.

### 2.3 Animals

Male ICR mice (weight, 12–14 g) and male Sprague–Dawley (SD) rats (weight, 230–250 g) were purchased from Beijing Huafukang Biotechnology Co. Ltd. (certificated no. 11401300087854 and 1103221911001951). All animals were acclimated for 1 week at a temperature of 24°C ± 1°C and humidity of 55% ± 5%. All animals were housed in cages with food and tap water ad libitum. All experiments were performed according to the guidelines specified in the Good Laboratory Practice Regulations by China Food and Drug Administration (CFDA) and recommendations of the National Institutes of Health Guide regarding the Care and Use of Laboratory Animals. The permission of animal use was approved by Office of Experimental Animal Management Committee of Shandong Province, China (License number: SYXK [Lu] 20230017).

### 2.4 Synthesis and Characterization of ApoE‐Mimetic Peptide

**FIGURE 1:**
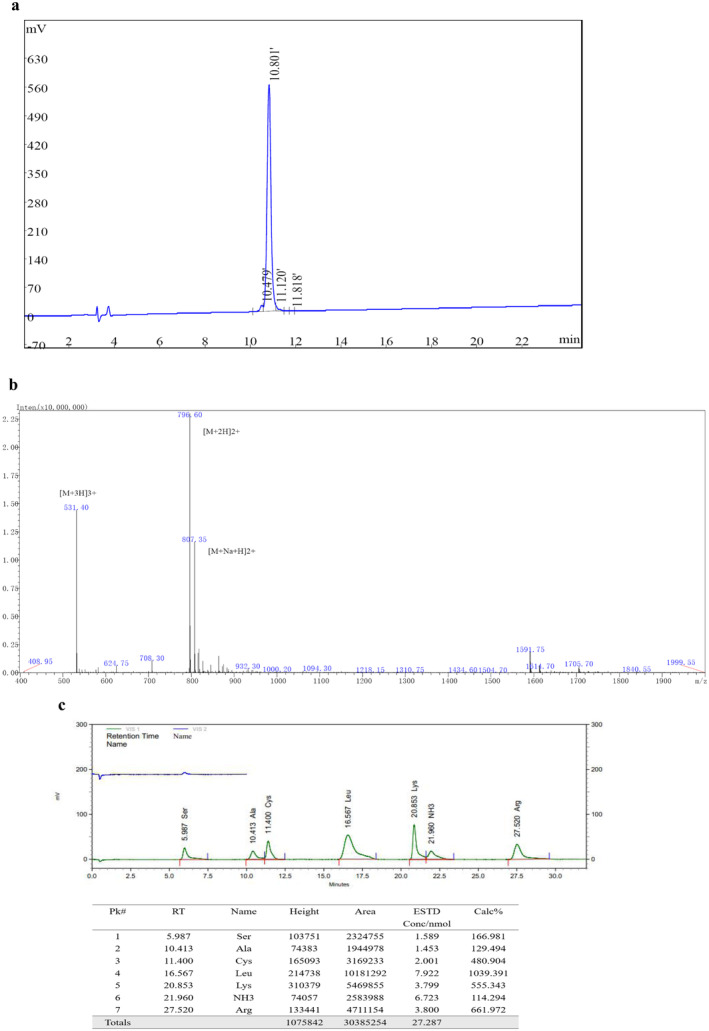
Characterization of CS15. (a) HPLC; (b) mass spectrometry; and (c) amino acid composition.

The sequence of the peptide is picolinoyl‐AS‐cyclo[Cys‐LRKL‐Aib‐KRLL‐Cys]‐NH2, which was provided by Cornerstone Therapeutics (Shanghai) Ltd. and designed by Dr. Yuchen Chen and Dr. Fengqiao Li. The new peptide is termed as CS15, which mimics the ApoE receptor binding domain from residues 138 to 149. CS15 was synthesized from GenicBiotechnology Co. Ltd. (Shanghai, China), using Fmoc solid‐phase synthesis method on rink amide AM resin. Fmoc‐protected amino acids (0.42 mmol) were coupled sequentially. Each reaction was allowed for 30 min. Fmoc deprotection was performed with 20% piperidine/DMF solution (DBLK) for 15 min. After coupling all fragments, peptide resins were washed with DMF, DCM, and methanol, respectively, and washed three times with each. Peptide was cleaved from the resin with trifluoroacetic acid (TFA), washed with diethyl ether, and precipitated. After obtaining the linear peptide, the cyclization was performed in acetic acid solution by dropwise addition of iodine‐methanol (10 mol/L) for oxidation. Finally, the crude cyclic peptide was then purified and exchanged to acetate salt by reverse‐phase high performance liquid chromatopraphy (HPLC) on a column of ChromCore 120 C18 5 μm. The peak fractions collected were characterized by ionization mass spectrometry. The purity of CS15 was 96.39% and MS (ESI) *m*/*z*: 531.40 [M + 3H]^3+^, 796.60 [M + 2H]^2+^ (Figure 1a,b). The amino acid composition analyzed by Hitachi L‐8900 Amino Acid Analyzer was shown in Figure 1c.

### 2.5 Measurement of TNF‐α and NO Production In Vitro

Cell viability was tested by using CCK‐8 assay before conducting the following test. BV2 cells were seeded in a 96‐well plate at the density of 4 × 10^5^ cells/mL overnight. The cells were pre‐treated with or without CS15 or COG1410 (0.03–9 μM) for 45 min and followed with LPS (0.2 μg/mL) for 24 h. Twenty microliters of supernatant were collected for determination of the TNF‐α level. The TNF‐α level was assayed by using a commercial ELISA kit according to the manufacturer's instructions (70‐EK282/3‐96). Briefly, 100 μL (1:10 dilution) of cell culture supernatant was added into a 96‐well ELISA plate coated with anti‐TNF‐α antibody and incubated at room temperature for 2 h. After washing to remove unbound antibody, HRP‐labeled detection antibody was added and incubated for at room temperature for 45 min. The plate was washed and 100 μL of TMB solution was added. The reaction was terminated after approximately 5–10 min. The absorbance was read at 450 nm and 630 nm wavelength using a Synergy 4 microplate reader (BioTek). The TNF‐α concentration was calculated based on a standard curve.

The cells were sequentially cultured for another 24 h after supplement with fresh medium. The nitrite concentration in the supernatant represented as the NO production was measured by the Griess method. The cell supernatant was added into a 96‐well plate, with 50 μL per well. Then, 50 μL of Griess reagent B (5% H_3_PO_4_ containing 1% sulfonamide) was added to each well (including the standard curve well). After 10 min of light avoidance reaction at room temperature, 50 μL of Griess reagent A (0.1% N‐naphthylethylenediamine hydrochloride) was added to each well. After 10 min of light avoidance reaction at room temperature, the absorbance was measured at 520 nm wavelength within 30 min. A standard curve was drawn by using the standard working solution of NaNO_2_ (100, 50, 25, 12.5, 6.25, 3.13, 1.56, and 0 μM). The concentration of NO^2−^ in the cell culture supernatant and the inhibition rate of NO release was calculated based on the NaNO_2_ standard curve.

### 2.6 LPS‐Induced TNF‐α Release in Mouse

ICR mice were randomly assigned into the following groups (*n* = 7): Control, LPS alone (LPS), LPS plus Dex (1 mg/kg), LPS plus CS15 (0.125, 0.25, 0.5, 1, 2, 4, and 8 mg/kg), and LPS plus COG1410 (0.5, 0.75, 1.13, 1.69, 2.53, 3.8, 5.7, and 8.54 mg/kg). All mice were intravenously administered once with above drugs/tested materials. Mice in control and LPS‐only group were injected with the same volume of normal saline. Thirty minutes after the treatment, LPS (5 μg/kg) were given intravenously to mice to induce inflammation. Mice in control group were injected with normal saline instead. One half an hour after LPS injection, blood was collected from the inner canthus of mice and anticoagulated with EDTA·Na_2_. Collected blood was centrifuged at 5000 rpm for 15 min to prepared plasma. TNF‐α level in plasma was measured by using a commercial ELISA kit according to the manufacturer's instructions.

### 2.7 Preparation of Gold Nanoparticles and CS15‐Coated Gold Nanoparticles (CS15‐GNPs) and Their Physicochemical Characterization

GNPs were synthesized through the classical citrate reduction method [13]. In brief, 4.0 mL sodium citrate (1% w/v) was added into 100 mL boiling HAuCl_4_ (0.01% w/v). The reaction took place for 15 min until solution turned from blue to burgundy red, indicating the formation of GNPs. The solution was cooled down to room temperature and then characterized.

To modify the surface of GNPs with PEG, 1 mM GNPs was allowed to react with 1 mM SH‐PEG2000‐TK‐COOH (GNPs: SH‐PEG‐TK‐COOH = 20:1, v/v), under mild stirring at 400 rpm for 48 h. The mixture was centrifuged using hyperfiltration tube at 4700 rpm for 15 min to remove free polymers and dispersed with PBS. Then, 1‐ethyl‐3‐(3‐dimethylaminopropyl) carbodiimide (EDC)/N‐hydroxysuccinimide (NHS) was added to the mixture for activating the carboxylic groups and stirred at 400 rpm for 20 min. The mixture was centrifuged at 4700 rpm for 15 min to remove uncoupled by‐products and dispersed with PBS. Finally, CS15 (1 mg/mL) was added at a peptide‐to‐GNPs volume ratio of 1:20. The mixture was reacted for 10 h at room temperature under agitation, and re‐centrifuged using hyperfiltration tube at 4700 rpm for 15 min to remove uncoupled peptide, dispersed with PBS, culminating in the production of CS15‐coated GNPs (CS15‐GNPs). The fluorescent peptide‐coated GNPs (Cy5‐CS15‐GNPs) was made by using Cy5‐labeled CS15 to substitute for CS15.

To characterize CS15‐GNPs, the particle diameters and diameters distribution, as well as surface zeta potential, were determined by nanoparticle size analyzer (Beckman Coulter, Brea, CA, USA). The morphology of GNPs and CS15‐GNPs was observed by transmission electron microscopy (TEM) (JEOL, Tokyo, Japan). Ultraviolet–visible (UV–Vis) spectroscopy was conducted to detected absorption spectra using UV‐2450 spectrophotometer (Shimadzu, Kyoto, Japan).

The drug‐loading efficiency of GNPs was determined by HPLC (Dikma technologies, Foothill Ranch, CA, United States). HPLC was conducted using a ChromCore 120 C18 column (4.6 × 250 mm, 5 μm). The flow rate and column oven temperature were set to 1.0 mL/min and 25°C. The mobile phase consisted of 0.1% acetic acid in acetonitrile and 0.1% acetic acid in water. The detection wavelength was 220 nm. The drug‐loading efficiency was calculated by the following equation:

$$\begin{array}{c} \text{Drug}‐\text{loading}\;\left(\%\right)=\frac{\text{Total drug}-\text{free drug}}{\text{GNPs or conjugated}‐\text{GNPs}+\text{total drug}-\text{free drug}}\\ \;\times 100\% \end{array}$$

The in vitro drug release was analyzed by dialysis method. Samples were placed in the dialysis bays, and subsequently 20 mL of PBS as a release medium (containing 0.1% Tween 80, without or with H_2_O_2_ at concentration of 0.1 and 1 mM, respectively) was added into the bays. The reaction was lasted for 100 h at 37°C with continuous gentle stirring. At selected time points, 200 μL of solution was collected from the release medium, and the amount of CS15 was detected by HPLC. The cumulative release rate of CS15 was calculated.

### 2.8 Gold Quantification and Brain Distribution After Nasal Delivery

Inductively coupled plasma mass spectrometry (ICP‐MS) was used to quantified the amount of gold, in order to evaluate the accumulation of CS15‐GNPs in brain tissues. SD rats were intranasally instilled with GNPs and CS15‐GNPs, with five animals in each group. The dose of gold administered to rat was 50 μg Au/kg. The administered volume was 50 μL/rat. One and 24 h after single administration, all rats were sacrificed, and perfused with normal saline to eliminate residual blood from the brain. Brain was harvested, washed with saline and patted dry on the filter paper. A certain amount of cerebral cortex (1.0 g) was weighed, placed in the polytetrafluoroethylene digestion tank, and then immersed in 5 mL aqua fortis for 1 h. Thereafter, 2 mL of hydrogen peroxide was added into the digestion vessel. Then, all samples were digested by a microwave digestion instrument (ETHOS One, MileStone, Italy) at 180°C for 1 h. At the end of digestion, the digested products were heated to 140°C in an acid driving instrument (LabTech VB20, LabTech, China) to drive off the vapor of nitrogen oxides until the acid volume reached about 0.5 mL. After cooling to room temperature, the final product was diluted with 2% HNO_3_ in a 25 mL volumetric flask for ICP‐MS measurement.

To visualize the distribution of CS15‐GNPs in the brain, tissue fluorescence images were analyzed. Rats were given intranasally with Cys‐CS15‐GNPs. Rats in control were given the same volume of water. One or 24 h after administration, rats were sacrificed, and the heart was perfused with saline to remove any remaining blood in the brains. Brains were collected and cut into slices, the fluorescence images were captured using the IVScope8000 spectrum live animal imaging system (CLINX, China), with excitation 640 nm and emission 670 nm.

### 2.9 Transient Middle Cerebral Artery Occlusion (MCAO) and Treatment Protocol

The surgical procedure was performed as described in our previous report [14]. Animals were anesthetized with 3% isoflurane and subsequently maintained anesthetic status with 2% isoflurane delivered with a face mask. Each rat was shaved and cleaned with a 75% alcohol swab. Rats were placed in a prone position and subjected to left MCAO. A midline incision was made at the neck to expose the left carotid region. The common carotid arteries (CCA), the external carotid arteries (ECA) and the internal carotid arteries (ICA) were carefully separated from the surrounding tissue, including the vagus nerve. A 2‐cm length of a 4–0 monofilament nylon suture with a silicon coated tip (diameter, 0.38 mm) was inserted through the small incision in CCA and further pushed along the lumen of ICA until resistance was felt, approximately 18 mm beyond the bifurcation of the CCA. After fixing the suture, the midline neck wound was infiltrated with 1% lidocaine and was closed. After that, animals were returned to their home cages and were allowed to regain consciousness. After 90 min of occlusion, the animals were anesthetized again and the filament was gently removed from ICA for reperfusion. Then the animals were placed in their normal habitat. Body temperature was maintained in the normal rang (36.5°C–37.5°C) with a heating pad during the operation. Temperature was monitored with a rectal probe. The sham‐operated animals were subjected to the same procedure without occlusion of MCA.

CS15 at dose of 0.1 mg/kg were administered by intravenous bolus immediately before reperfusion, and 3 h after reperfusion, and then at 24 and 48 h after reperfusion. CS15‐GNPs 20 and 40 μg/mL (50 μL per rat, CS15 dose = 3.5 and 7 μg/kg) were given once through intranasal (IN) route immediately before reperfusion. The same volumes of normal saline were administered in the same manner to rats of the sham and MCAO groups.

### 2.10 Neurological Evaluation and Infarct Analysis

The neurological evaluation was performed at 24, 48, and 72 h after reperfusion according to the method described by Yokoo et al. [15]. The total score is 48, which represents worst function. The score was performed by an observer blinded to group assignment. After the final neurological evaluation, animals were sacrificed with deeply anesthetized with ketamine (80 mg/kg, ip) and xylazine (10 mg/kg, ip). Then the brains were removed, immersed in normal saline to clean residual blood, and subsequently frozen at −20°C for 15–20 min. The brains were sliced into 2‐mm‐thick coronal sections (6 slices per brain) with the aid of a brain slicer matrix. Brain slices were incubated in freshly prepared 2% TTC solution for 10 min at 37°C under dark condition, after which stained brain sections were fixed in 4% paraformaldehyde solution for photography. Brain areas were traced and measured using the Image‐pro plus 6.0 software. The infarcted regions and the areas of both hemispheres were calculated for each brain slice. The average area of infarction was expressed as the percentage of the whole coronal section.

### 2.11 Immunohistochemical and Immunofluorescence Assay

Brain tissues were collected at 72 h after MCAO and fixed in 4% neutral paraformaldehyde. Immunostaining was performed by standard protocols. Briefly, paraffin‐embedded brain tissue sections (5 μm thickness) were regularly dewaxed and hydrated through graded ethanol. Sodium citrated solution was used for heat‐induced antigen retrieval. The sections were treated for 10 min with 3% H_2_O_2_ followed by 5% BSA to block nonspecific binding for 20 min. After that, sections were incubated with anti‐NeuN antibody at a 1:200 dilution overnight and then with a horseradish peroxidase (HRP)‐labeled goat anti‐rabbit IgG secondary antibody for 1 h at 37°C. The antibody signal was detected using DAB regents. Immunohistochemical images were obtained using OLYMPUS microscope BX53 (original magnifications × 200). Quantitative analysis was performed by counting the numbers of positively stained cells from five randomly selected microscopic regions in the ischemic hemispheres, which was analyzed using Image J software. Data were expressed as mean cell numbers per rat.

For iNOS/Iba1 immunofluorescence analysis, sections were incubated with antibodies of Iba1 (GB153502‐100, Servicebio) at a 1:1000 dilution and iNOS at a 1:500 (GB11119‐100, Servicebio) overnight, respectively, and then with an Alexa Fluor 488‐labeled goat anti‐rabbit IgG secondary antibody (1:300) and with a CY3‐labeled goat anti‐rabbit IgG secondary antibody (1:300) at room temperature for 50 min in the dark condition. After washing, slides were stained using a 4′,6‐diamidino‐2‐phenylindole (DAPI) medium. For TUNEL/NeuN immunofluorescent double‐labeled staining, brain slices were incubated with anti‐NeuN antibody (at a 1:500 dilution) and then incubated with TUNEL test regent. The FTIC‐labeled goat anti‐rabbit IgG secondary antibody (1:1000) was employed to detect antibody signal. Finally, the slides were mounted with mounting medium containing fluorescence anti‐quenching agent and coverslip. The slices were scanned by panoslice scanner (PANNORAMIC DESK/MIDI/250/1000, 3DHISTECH, Hungary). Fluorescence images for TUNEL/NeuN double‐labeled staining were obtained using OLYMPUS fluorescence microscope BX53 (original magnifications × 100). Three randomly selected microscopic regions per section were imaged and iNOS/Iba1‐positive cells count was performed using Image J software, Data were expressed as mean cell numbers per rat. In addition, we counted total NeuN‐positive cells and TUNEL/NeuN‐positive cells to calculate the percentage of apoptotic neurons.

### 2.12 Measurement of TNF‐α and IL‐6 Levels in Brain Tissues

Brain tissues were collected at 72 h after MCAO. About 0.2 g brain tissue in the ischemic hemispheres were homogenized on ice in PBS buffer to prepare a 10% homogenate. The homogenate was immediately centrifuged at 12000 rpm for 10 min at 4°C. The supernatant was collected for the measurement of TNF‐α and IL‐6 levels using ELISA kits. The process of assay was performed according to the manufacturer's instructions. The final results were expressed as pg/mg protein.

### 2.13 Biosafety and Biocompatibility Evaluation of CS15‐GNPs

Fifteen rats were randomly divided into three groups: Control, GNPs, and CS15‐GNPs (40 μg/mL, 50 μL, and 7 μg/kg) with five rats in each group. Animals were administered intranasally with C15‐GNPs, once daily for 7 days. The same volumes of naked GNPs were administered in the same manner to rats. Rats in control were given same volumes of normal saline. Animals were observed for changes in clinical signs and toxic reactions. At the end of administration, blood was collected for serum biochemistry from abdominal aorta. Then, animals were dissected and visual observation were performed. Key organs including liver, spleen, lung, and nasal septum with the epithelial cells were collected. All samples were fixed in 4% neutral buffered formalin, decalcified and dehydrated with ethanol, paraffin embedded, and were sliced into 5 μm sections. Sections were stained with H&E and followed microscopic examination.

### 2.14 Statistical Analysis

Statistical analysis was performed using GraphPad_Prism software 7.0 for Windows. All results were expressed as mean ± standard deviation (SD). Test for normality of data was Shapiro–Wilk normality test. Quantitative data were tested for homogeneity of variance. If the variances were equal (*p* > 0.05), one‐way ANOVA followed by Dunnett test was used. If the variances were unequal (*p* < 0.05). Nonparametric tests were performed. Comparisons between groups were made using the Kruskal–Wallis test followed by the Dunn's multiple comparisons test. *p* < 0.05 was considered significant.

## 3 Results

### 3.1 CS15 Exerted Potent Anti‐Inflammation in LPS‐Induced Inflammatory Response In Vitro and In Vivo

**FIGURE 2:**
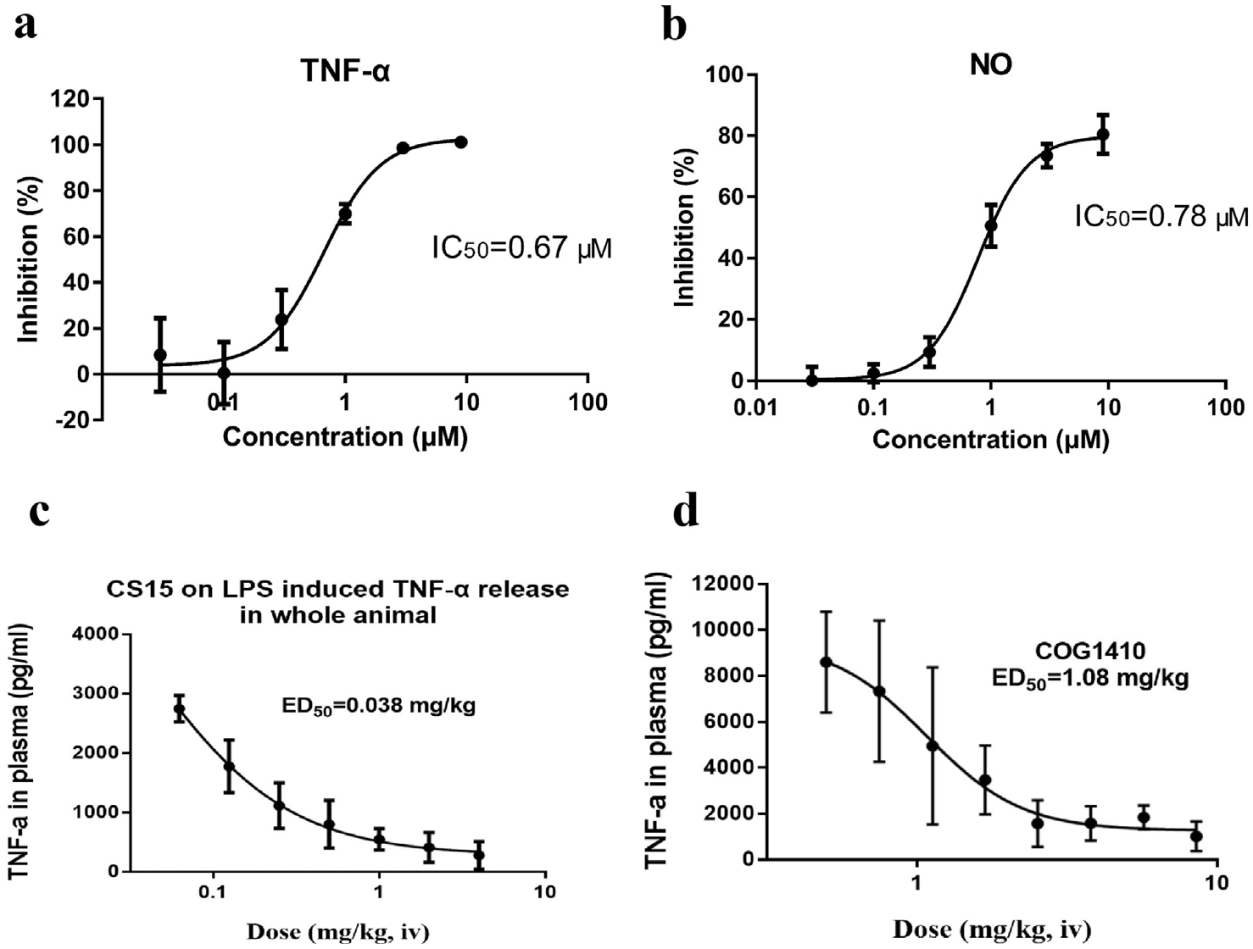
CS15 inhibited TNF‐α and NO production in vitro and in vivo. BV2 cells were treated with 1 μg/mL of LPS with or without CS15 or COG1410 (0.03, 0.1, 0.3, 1, 3, and 9 μM) for 24 or 48 h. The supernatant was taken out for the measurement of TNF‐α and NO production using ELISA and Griess method, respectively. BALB/c mice were intravenously administered once with Dex (10 mg/kg), CS15 (0.125, 0.25, 0.5, 1, 2, 4, and 8 mg/kg), COG1410 (0.5, 0.75, 1.13, 1.69, 2.53, 3.8, 5.7, and 8.54 mg/kg). Thirty minutes after the treatment, LPS (5 μg/kg) were given intravenously to mice to induce inflammation. One and half hours after LPS injection, blood was collected to prepared plasma. TNF‐α level in plasma was measured using ELISA kit. (a) Inhibition on TNF‐α; (b) Inhibition on NO; (c) TNF‐α inhibition of CS15; (d) TNF‐α inhibition of COG1410. Data are represented as mean ± SD of six animals of each group.

In our previous study, CS15 at concentration of 10 μM did not show cytotoxicity against BV‐2 cells. Therefore, this dose range below 10 μM was used in the following experiment. BV2 cells were treated with 0.2 μg/mL of LPS with or without CS15. As shown in Figure 2a,b, CS15 suppressed the production of TNF‐α and NO in a concentration‐dependent manner. The 50% inhibitory concentrations (IC50) were 0.67 and 0.78 μM, respectively. The IC_50_ value of COG1410 on TNF‐α inhibition was 4.5 μM. CS15 exhibited more potent anti‐inflammation than COG1410.

Furthermore, we used LPS‐induced systemic inflammatory response to explore the anti‐inflammation of CS15 in vivo. As shown in Figure 2c, CS15 markedly decreased TNF‐α production in a dose‐dependent manner. The median effective dose (ED_50_) was 0.038 mg/kg. The ED_50_ value of COG1410 was 1.08 mg/kg under the same experimental condition (Figure 2d). The potency of CS15 is superior to that of COG1410. Dex 1 mg/kg demonstrated approximately 100% inhibition on TNF‐α production.

### 3.2 Characterization of GNPs and CS15‐GNPs

**FIGURE 3:**
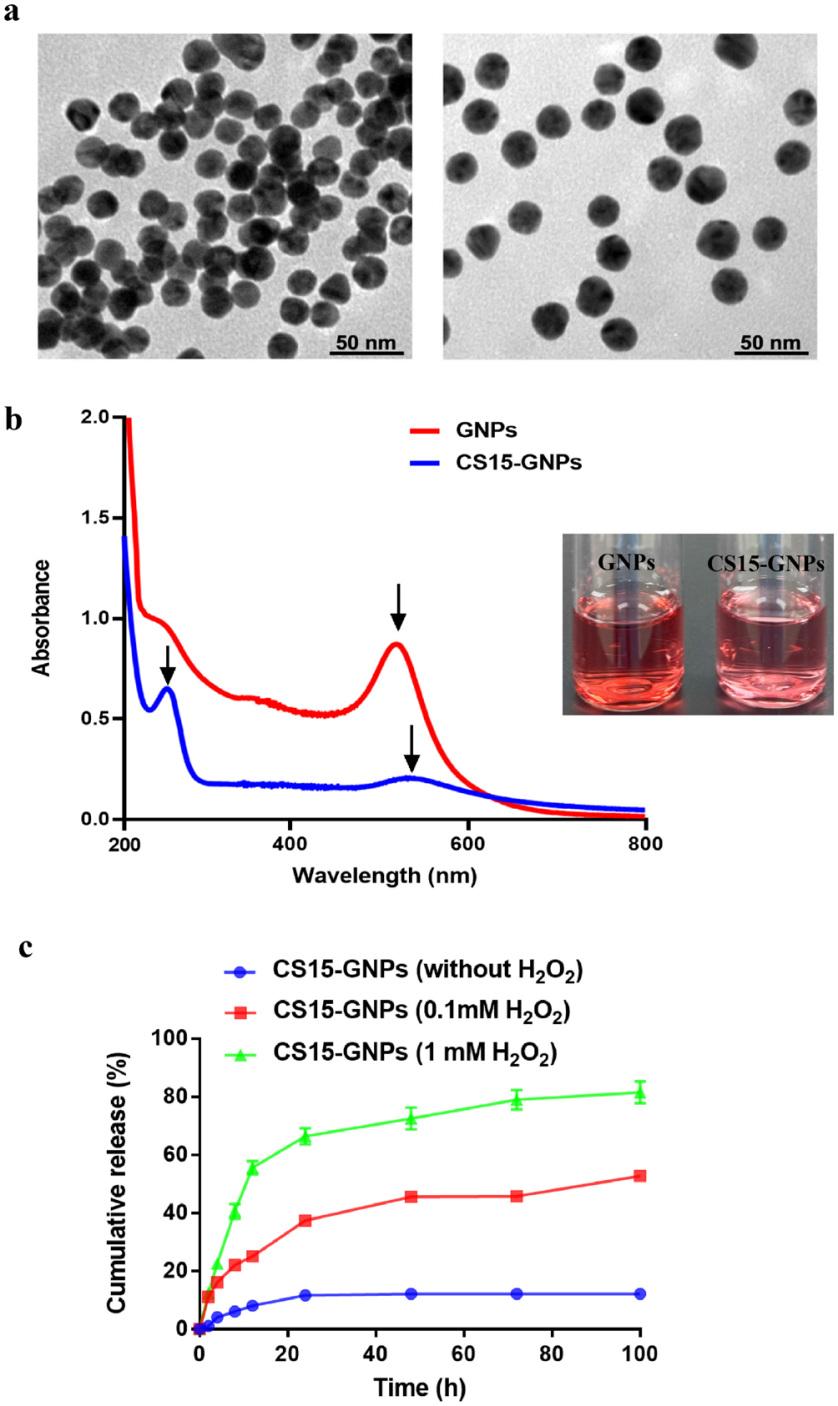
Characterization of GNPs and CS15‐GNPs. (a) Representative TEM images of GNPs and CS15‐GNPs. Scale bars, 50 nm. (b) UV–Vis absorbance spectra of GNPs and CS15‐GNPs. Arrows indicate absorption peaks. (c) The cumulative release curves of CS15‐GNPs.

The shape and surface morphology of prepared GNPs and CS15‐GNPs were imaged by TEM. As shown in Figure 3a, GNPs showed a typical spherical morphology with uniform particles size. The GNPs were covalently modified with sulfur‐gold bonds by SH‐PEG‐COOH and then connected with CS15 to form CS15‐GNPs. CS15‐GNPs still exhibited a typical spherical morphology and had a better particle dispersion based on TEM images. The UV–Vis spectra showed that GNPs had a typical UV absorption peak at 519 nm. The absorption peak of CS15‐GNPs was red shifted to 534 nm, and a new typical absorption peak appeared at 261 nm (Figure 3b), which indicated that CS15 was successfully loaded onto GNPs. The properties of the GNPs and CS15‐GNPs are summarized in Table 1. The average of diameter of GNPs was ~15 nm and increased to ~40 nm after surface modification. A negative surface charge was −28.4 ± 1.4 mV for GNPs, which was changed to −4.46 ± 1.6 for CS15‐GNPs. Drug loading of CS15‐GNPs was determined to be 23.01% ± 0.29%.

**TABLE 1:** Physicochemical properties of bare GNPs and CS15‐GNPs.

Formulation	Particle size (nm)	Polydispersity index	Zeta potential (mV)
GNPs	15.45 ± 1.3	0.18 ± 0.04	−28.4 ± 1.4
CS15‐GNPs	38.12 ± 1.7	0.22 ± 0.05	−4.46 ± 1.6

Given that inflammation is a crucial factor in the pathophysiology of acute ischemic stroke, we constructed a ROS responsiveness of CS15‐GNPs in which SH‐PEG2000‐TK‐COOH served as a linker that contains a ROS‐responsive thioketal structure. To verified the drug release behavior of CS15‐GNPs in the inflammatory microenvironment, the drug release ratio of CS15 was determined at different concentrations of H_2_O_2_. As shown in Figure 3c, about 80% of CS15 was released at higher H_2_O_2_ concentrations (1 mM) after incubation 72 h. In contrast, only 12.1% of CS15 was released in PBS solution without H_2_O_2_. This indicated that inflammatory environment during ischemia injury contributes to efficient release of CS15, and CS15‐GNPs maintains stable in physiological environment.

### 3.3 Quantitative and Qualitative Analysis of Gold Nanoparticle Accumulation in Brain After IN Administration

To evidence GNPs transport and accumulation in the brain, gold quantification was carried out by ICP‐MS method, whereas qualitative estimation was performed by fluorescence.

**FIGURE 4:**
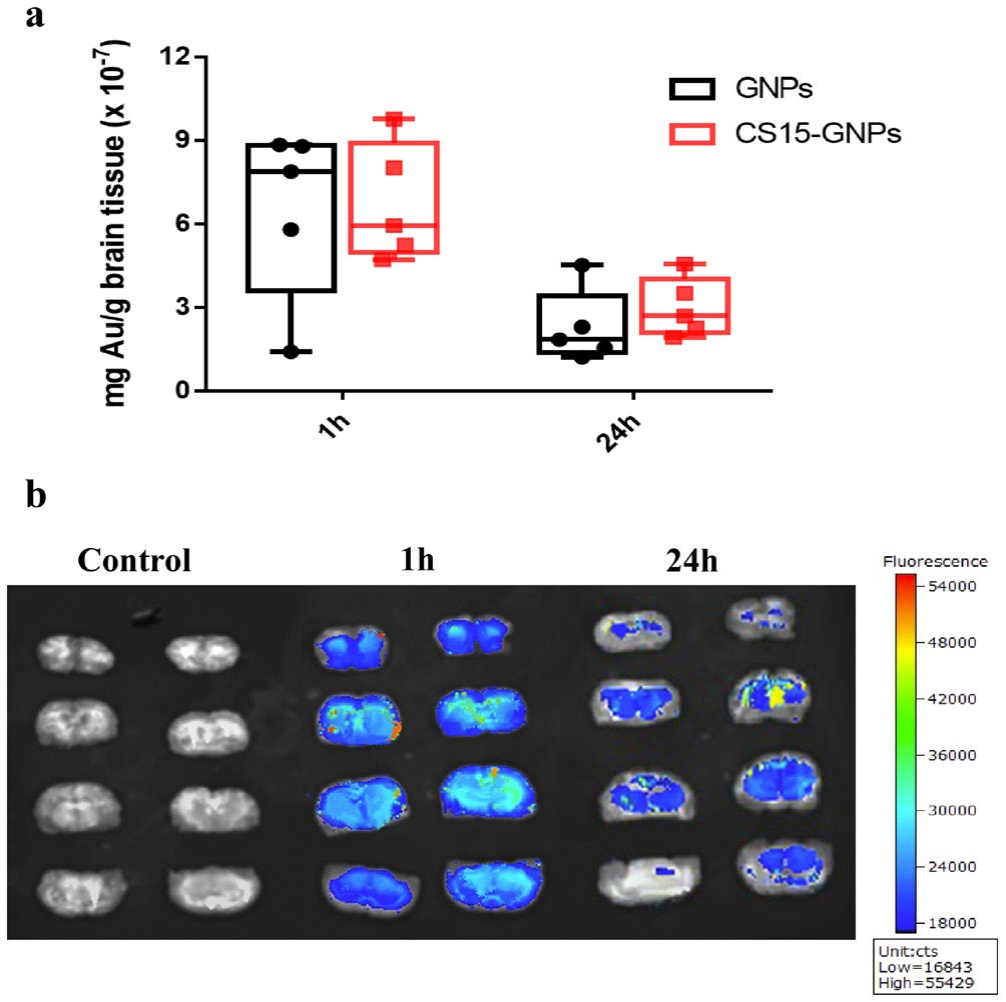
Quantitative and qualitative analysis of gold nanoparticle accumulation in brain. (a) Gold quantitation by ICP‐MS. (b) Fluorescence images of coronal brain sections of rat. Cy5‐CS15‐GNPs were administered to rats by intranasal administration. One or 24 h after administration, rats were sacrificed, and perfused with saline to remove the blood residues. Brains were removed and cut into slices, the fluorescence images were captured using the IVScope8000 spectrum live animal imaging system, with excitation 640 nm and emission 670 nm.

The gold level of brain was determined at 1 and 24 h after intranasal administration. As shown in Figure 4a, there was no significant difference between the amount of gold found in GNPs [6.53 ± 3.12 mg/g tissue (×10^−7^) at 1 h and 2.28 ± 1.32 mg/g tissue (×10^−7^) at 24 h] and CS15‐GNPs [6.73 ± 2.11 mg/g tissue (×10^−7^) at 1 h and 2.98 ± 1.06 mg/g tissue (×10^−7^) at 24 h], indicating that peptide modification had no influence on the cell uptake of gold nanoparticles. The fluorescence images further verified the distribution of gold in the brain and fluorescence intensity could be detected until 24 h after administration (Figure 4b), which was consistent with quantitative result. These results suggested that GNPs are capable of delivering CS15 to the brain, and sustained releasing CS15.

### 3.4 Neuroprotective of CS15‐GNPs in MCAO in Rats

**FIGURE 5:**
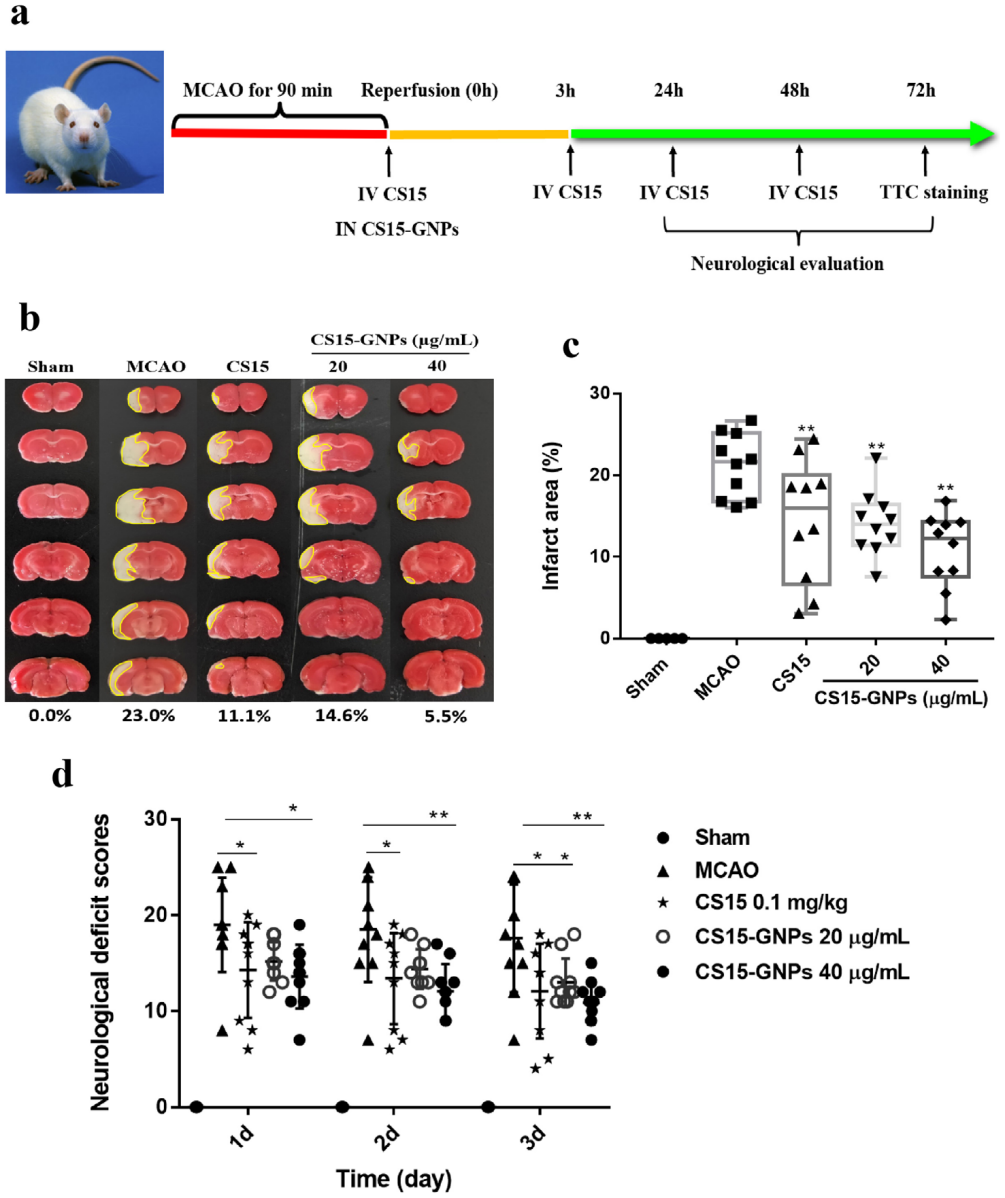
Neuroprotective of CS15‐GNPs in MCAO in rats. Rats were intravenously administered CS15 0.1 mg/kg and CS15‐GNPs 20 and 40 μg/mL (3.5 and 7 μg/kg) and after MCAO surgery. The frequency of administration was described in method section. (a) The schematic of the experiment design; (b) Representative coronal sections of each group; (c) Infarct area of each group; (d) Neurological scores. Data are represented as mean ± SD of 10 animals of each group (*n* = 5 for sham). *p < 0.05 and **p < 0.01 versus MCAO group. The areas outlined by the yellow line indicate infarcted areas.

Representative sections of each group are presented in Figure 5b. As shown in Figure 5c, the infarct size of MCAO group was 21.2% ± 3.9% at 72 h after reperfusion. Treatment with CS15 0.1 mg/kg resulted in 32.1% reduction in infarct size, which was significantly decreased as compared with MCAO group. Similarly, nasal administration of CS15‐GNPs 20 and 40 μg/mL exerted excellent decreases in infarct size. The infarct size was 14.1% ± 4.0% and 10.8% ± 4.6%, which represented 33.5% and 49% reduction in infarct size, respectively. The effect of CS15‐GNPs 20 μg/mL is equivalent to that of CS15 0.1 mg/kg. CS15‐GNPs 40 μg/mL showed the most potent effect.

In addition, administration of CS15 and CS15‐GNPs 40 μg/mL markedly reduced neurological scores at all selected time points. CS15‐GNPs 20 μg/mL showed significant improvement of neurological symptoms on days 2 and 3 (Figure 5d).

### 3.5 Effect of CS15‐GNPs on Ischemic Neuronal Injury

**FIGURE 6:**
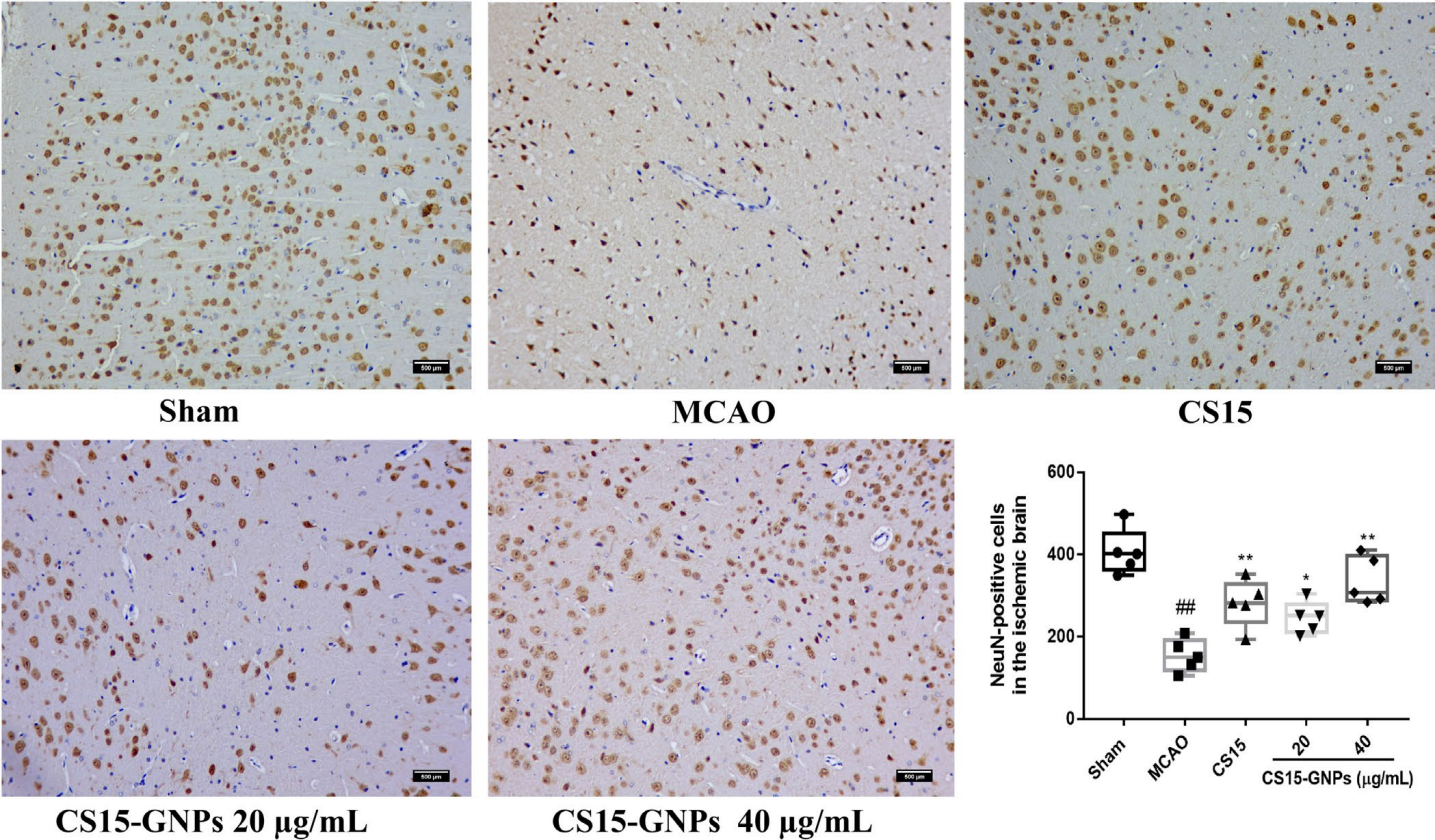
CS15‐GNPs alleviated neuron loss. Representative image of NeuN staining and NeuN‐positive cells (Original magnification, ×200; scale bar = 500 μm). Data are represented as mean ± SD of 5 animals of each group. ^##^ *p* < 0.01 versus Sham, *p < 0.05, **p < 0.01 versus MCAO group.

**FIGURE 7:**
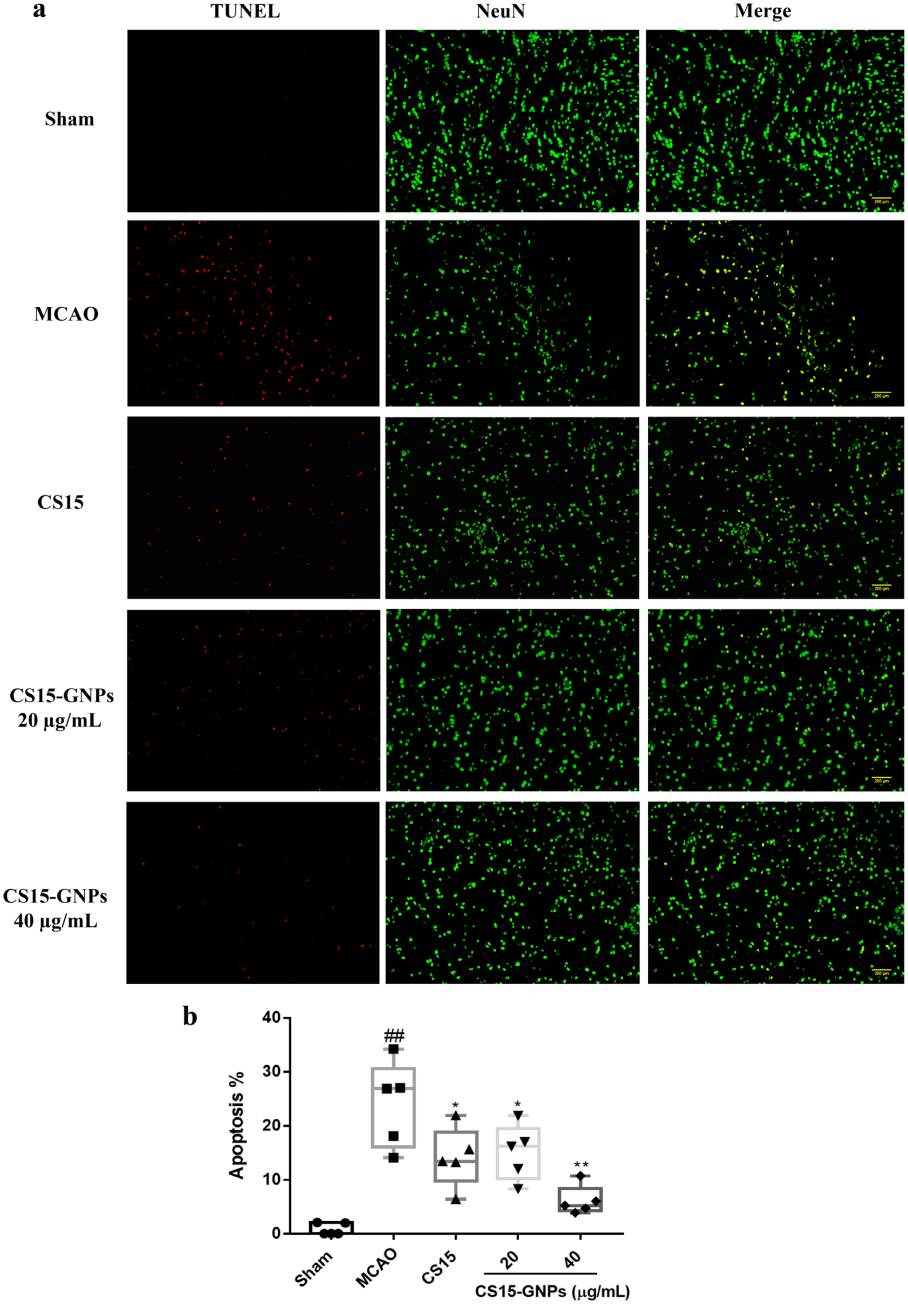
CS15‐GNPs reduced neuronal apoptosis. (a) Immunofluorescent pictures of TUNEL/NeuN double‐labeled staining of brain tissues (original magnification, ×100; scale bar = 200 μm); (b) The percentage of number of TUNEL/NeuN‐positive cells. Data are represented as mean ± SD of five animals of each group. ^##^ *p* < 0.01 versus Sham, *p < 0.05, **p < 0.01 versus MCAO group.

The results showed that the number of neuron‐specific marker NeuN‐positive cells significantly decreased in brain tissue at 72 after ischemia–reperfusion (Figure 6), accompanied with increase in neuronal apoptosis (Figure 7), which indicated the loss of neurons induce by ischemic stroke. Intranasal administration of CS15‐GNPs 40 μg/mL resulted in markedly higher number of neurons and reduced the number of apoptotic cells. CS15‐GNPs 20 μg/mL and CS15 showed similar effect (Figures 6 and 7). The result indicated that CS15‐GNPs showed better protective effect against neuronal damage than free CS15.

### 3.6 Effect of CS15‐GNPs on Cerebral Inflammation

**FIGURE 8:**
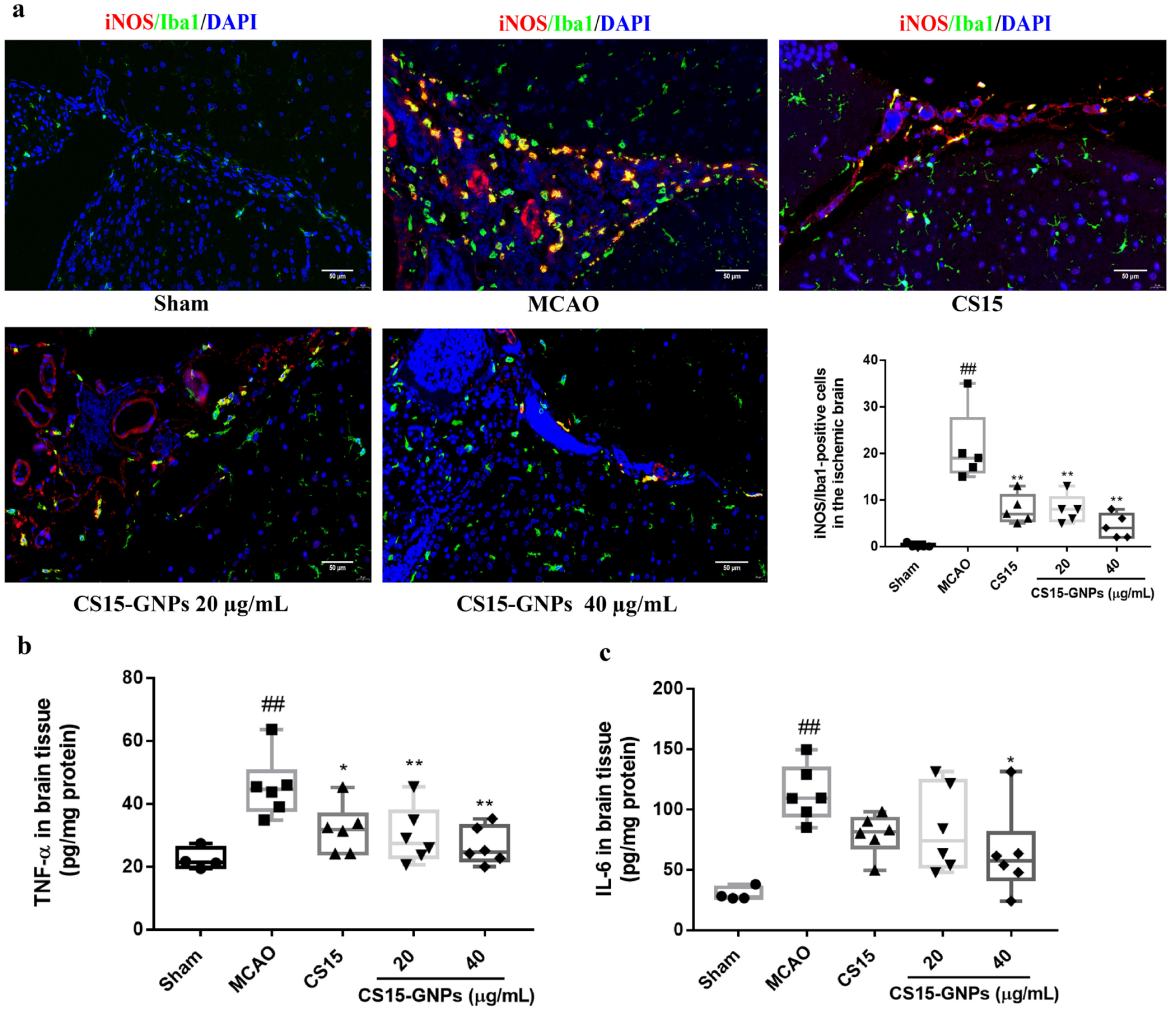
CS15‐GNPs ameliorated cerebral inflammation. (a) Immunofluorescence analysis of iNOS/Iba1 expression and iNOS/Iba1‐ positive cells (scale bar = 50 μm); (b and c) TNF‐α and IL‐6 levels in brain tissue. Data are represented as mean ± SD of five or six animals of each group. ^##^ *p* < 0.01 versus Sham, *p < 0.05, **p < 0.01 versus MCAO group.

As shown in Figure 8a, at 72 h after ischemia–reperfusion injury, there was a significant increase in the intensity and number of iNOS/Iba1‐stained cells in brain tissue of MCAO rats, especially in the periventricular areas. Administration of CS15 and CS15‐GNPs markedly reduced the intensity and number of iNOS/Iba1‐positive cells. Furthermore, CS15‐GNPs 40 μg/mL significantly decreased the production of TNF‐α and IL‐6. The TNF‐α level was markedly decreased in CS15 and CS15‐GNPs 20 μg/mL groups, whereas the reduction in IL‐6 level in both groups was not found to be statistically different compared to MCAO group (Figure 8b,c).

### 3.7 Seven Days Repeated Dose Toxicity Study of CS15‐GNPs in Rats

**FIGURE 9:**
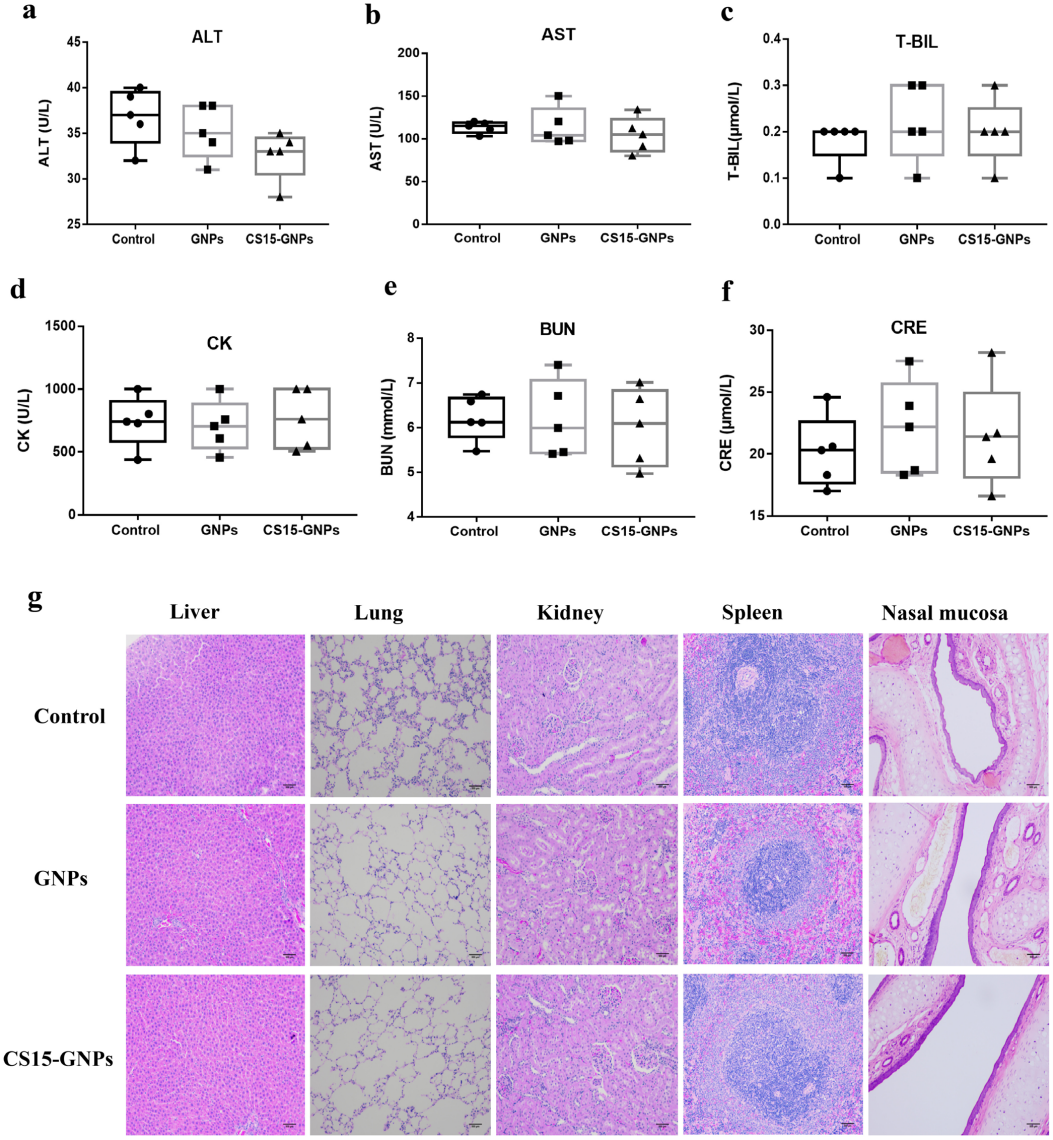
Biosafety of CS15‐GNPs. (a–f) Effects of CS15‐GNPs on clinical chemistry of rats. (g) Representative images of histopathological examination showed no significant abnormalities in liver, lung, kidneys, spleen and nasal cavity in rats treated with CS15‐GNPs for 7 days compared with normal control (200× magnification; scale bar = 500 μm). Data are represented as mean ± SD of five animals of each group. ALT, alanine aminotransferase; AST, aspartate aminotransferase; BUN, blood urea nitrogen; CK, creatine kinase; CRE, creatinine; T‐BIL, total bilirubin.

During the experiment, daily treatment with CS15‐GNPs for 7 days had found no clinical abnormalities in animals. The main parameters of blood chemistry did not significantly differ in animals in GNPs, CS15‐GNPs, and control groups (Figure 9a–f). In addition, no macroscopic differences and significant histopathological abnormalities were observed in key organs (Figure 9g). The microscopic examination of nasal mucosa showed an intact tissue architecture and mucosal epithelial cell. No significant degenerative changes and inflammatory infiltration were observed.

## 4 Discussion

CS15 is a derived product of COG1410, with special chemical modification on C‐terminal and cyclization through Cys disulfide bonds, to improve structural stability and bioactivity. The sequence of COG1410 is Ac‐AS(Aib)LRKL(Aib)KRLL‐NH2, a 12‐aminoacid peptide. Previous studies have revealed that COG1410 improved vestibulomotor and sensorimotor functions in TBI mice [16]. It reduced infarct size and neuroinflammation, improved neurological function in animal models of ischemic stroke and subarachnoid hemorrhage [10, 17]. In the present study, we first employed LPS‐induced cytokine release in BV2 cells and in mice as method for anti‐inflammatory activity screening. Inflammatory response occurs after the ischemic event and persists throughout the entire process of occurrence and progression of stroke, which may serve as a valid target for pharmacological intervention [18, 19, 20]. Amelioration of brain inflammation and inflammation‐associated brain injury or modulation of brain inflammation remains as a therapeutic target of professionals and pharmaceutical colleagues [18, 19]. TNF‐α and NO are two well‐known cytokines implicated in the evolution of brain damage after an ischemia/reperfusion injury [18, 21], which are widely used in the evaluation of anti‐inflammation of tested articles in vitro. The results demonstrated that CS15 possessed potent anti‐inflammation in vitro and in vivo. The potency is superior to that of COG1410, which is worth further in‐depth research.

It is well‐known that peptide drugs tend to have a relatively short half‐life, which are easily destroyed and rapidly cleared in the body, leading to its inferior efficacy. Despite the structurally modified CS15 has shown improved biological activities, no significant improvement in biological stability was observed. In our previous study, intravenous administration of CS15 was rapidly eliminated from plasma in rats. The elimination half‐life (*t*_1/2_) of CS15 was approximately 5–6 min in rat, leading to insufficient amount of drug reaching the target organ. Thus, we need to develop a novel delivery approach for success peptide delivery. Theoretically, CS15 can cross the BBB to reach the brain through low‐density lipoprotein receptor (LDLR) and LDLR‐related protein 1 (LRP1) expressed in the brain endothelial cells. Therefore, a drug delivery strategy for carrying CS15 into CNS could not only prevent CS15 from degradation to extend the duration of action, but also not discount the cell penetration of CS15. GNPs typically have small size ranging from 10 to 100 nm, allowing them to penetrate the BBB to reach the brain more efficiently [22, 23]. In addition, it has been reported that GNPs own important anti‐inflammatory properties. Intra‐articular administration of GNPs inhibited joint inflammation and development of polyarthritis [24]. GNPs have been demonstrated to reduce neuroinflammation [25], improve neurologic deficits and exert neuroprotective effects in AD, PD and ischemic stroke models [26, 27, 28]. These make GNPs an ideal carrier for delivering neuroprotective agents into the brain for the treatment of cerebral inflammatory diseases. The present study described the preparation and efficacy of surface modification of GNPs with CS15. On the one side, small‐sized GNPs can transport across the BBB and be taken up by neurocytes. On the other side, coating GNPs with CS15 was expected to enhance the ability of anti‐neuroinflammation of CS15. The current result showed that we have successfully prepared CS15‐GNPs formulation, which possessed favorable physicochemical properties and release behavior in the inflammatory microenvironment.

The presence of blood–brain barrier limits therapeutic agents to enter into CNS. Different administration routes influence the brain uptake of nanoparticles. GNPs, like other type of nanoparticles, have limited ability to cross the BBB and reach the brain after intravenous injection. IN administration is one available solution of bypassing the BBB [29]. It is reported that IN route is more effective for targeting GNPs to the brain than IV administration. The level of gold in brain after IN administration was approximately 100‐fold higher compared to IV administration [30]. In addition, protease in blood may degrade the peptide cargoes when CS15‐GNPs are given intravenously, leading to suboptimal amount of peptide to reach the brain. Thus, we selected IN pathway to deliver the gold formulation. To evidence GNPs transport and accumulation in the brain, gold quantification and qualitative estimation was carried out. These results suggested that GNPs are capable of delivering CS15 to the brain, and sustained releasing CS15.

In order to evaluate the neuroprotective of CS15‐GNPs, MCAO model in rats was established. In the present experiment, CS15‐GNPs was given through nasal route because of its feasibility and more efficient delivery of drug to the brain [30, 31]. The dosage of CS15 was determined to be 0.1 mg/kg because it was the optimal dose in our previous studies. In this experiment, free CS15 0.1 mg/kg was less potent than CS15‐GNPs 40 μg/mL in improving neurological outcomes. Intranasal administration of CS15‐GNPs generated surprising outcome, which indicated that the GNPs as a carrier not only minimizes CS15 dose and prolongs the duration of action, but also enhances therapeutic effectiveness. The dose‐effect relationship of CS15‐GNPs and its long‐term neuroprotection remain to be determined in the future work.

Furthermore, we evaluated the effect of CS15‐GNPs on neuronal injury. Neurons participate in maintaining and stabilizing the completeness of brain function. Neuronal damage caused by neuron loss, oxidative stress and inflammatory response after ischemic stroke results in cerebral functional deficits. To improve neurological functional deficits by rescuing neuronal damage is considered to be a therapeutic goal for ischemic stroke [32]. The result showed that CS15‐GNPs inhibited neuron loss and neuronal apoptosis, which showed better protective effect against neuronal damage than free CS15.

Microglia cells are the resident macrophages of CNS, which play a dual role in CNS repair. In the injured brain, microglia clear cell debris and take part in neuronal restorative. However, activated microglia may hinder neural repair and even aggravate brain injury [33, 34]. Thus, suppression of microglia contributes to temper cerebral inflammation. Iba1 is an extensively used microglial marker. Following ischemic stroke, Iba1 expression is upregulated in activated microglia and Iba1‐labled microglia accumulate in the infarct area [35]. Activated microglia are classified into “detrimental” M1 phenotype and “protective” M2 phenotype. M1 microglia release proinflammatory cytokines, such as IL‐6, TNF‐α, and iNOS. iNOS can be used as a marker of M1 microglia [36]. The current result showed that CS15‐GNPs could reduce the expression and the number of iNOS/Iba1‐positive cells, as well as TNF‐α and IL‐6 levels. The result suggested that CS15 and CS15‐GNPs ameliorated neuroinflammation via inhibition of activation of microglia and release of proinflammatory cytokines. CS15‐GNPs formulation exhibited greater anti‐neuroinflammation effect than free CS15.

To further verify the toxicity of CS15‐GNPs after nasal administration, we conducted a 7‐day repeated dose toxicity test in rats. Daily treatment with CS15‐GNPs for 7 days had found no clinical and histopathological abnormalities in key organs as well as nasal mucosa. Those results confirmed the safety and biocompatibility of intranasal administration of CS15‐GNPs to target the brain. Certainly, the safety of long‐term administration of higher dosage of CS15‐GNPs remains to be determined.

## 5 Conclusion

In summary, the current study demonstrated that CS15 exerted more potent anti‐inflammation than COG1410 in vitro and in vivo. Gold nanoparticles was capable of delivering CS15 to the brain, expanding its duration of action and increasing activity. Intranasal administration of CS15‐GNPs showed favorable neuroprotection with higher efficiency than CS15, and had good biosafety. These findings provide pharmacological evidence for the application of CS15 for treatment of ischemic stroke. In the future, the systemic toxicity of CS15 and its gold formulation are need to perform. The relationship of pharmacokinetics (PK) and pharmacodynamics (PD) of CS15‐GNPs remains to be explored in order to promote clinical translation of CS15.

## Author Contributions

M.‐Y.Y.: conducted research, analyzed data and wrote original draft. Y.‐W.Y., T.L., Z.‐X.W., and B.‐F.G., X.‐X.B., and Y.‐P.H.: conducted research and performed statistical analysis. D.‐L.L.: conducted pathological analysis; H.‐Y.L.: performed statistical analysis; H.‐Y.F.: designed research and reviewed and edited article. All authors read and approved the final manuscript.

## Ethics Statement

All experiments were performed according to the guidelines specified in the Good Laboratory Practice Regulations by China Food and Drug Administration (CFDA) and recommendations of the National Institutes of Health Guide regarding the Care and Use of Laboratory Animals. The permission of animal use was approved by Office of Experimental Animal Management Committee of Shandong Province, China (License number: SYXK [Lu] 20230017).

## Conflicts of Interest

The authors declare no conflicts of interest.

## Data Availability

The datasets used and/or analyzed during the current study are available from the corresponding author upon reasonable request.
